# Initial Outcomes from a Minimally Invasive Cardiac Surgery—Off-Pump Coronary Artery Bypass Grafting (MICS-OPCAB) Programme: A Case Series of the First 50 Patients Single-Centre Experience

**DOI:** 10.3390/jcdd12120456

**Published:** 2025-11-25

**Authors:** Omar AlMawajdeh, Bilal H. Kirmani, Haytham Sabry, Andrew D. Muir

**Affiliations:** Department of Cardiothoracic Surgery, Liverpool Heart & Chest Hospital NHS Trust, Thomas Drive, Liverpool L14 3PE, UK; omar.al-mawajdeh@lhch.nhs.uk (O.A.); bil.kirmani@lhch.nhs.uk (B.H.K.); haytham.sabry@lhch.nhs.uk (H.S.)

**Keywords:** MICS-OPCAB, minimally invasive cardiac surgery, coronary artery bypass grafting, LIMA, postoperative outcomes

## Abstract

Background: Minimally invasive off-pump coronary artery bypass grafting (MICS-OPCAB) offers potential advantages over conventional sternotomy, including reduced trauma and faster recovery. This study evaluates the safety and feasibility of MICS-OPCAB at our centre. Methods: We retrospectively analysed 50 consecutive MICS-OPCAB procedures performed via left anterior thoracotomy at our institution between January 2023 and June 2025. Data collected included patient demographics, operative details, and postoperative outcomes. Endpoints were 30-day mortality, conversion to sternotomy, and postoperative complications. Results: The cohort included 41 males (82%) with a mean age of 63.1 ± 8.7 years (range 40–80) and mean BMI 27.8 ± 4.3 kg/m^2^. Comorbidities included diabetes mellitus in 26%, COPD in 12%, and chronic kidney disease in 8%. Canadian Cardiovascular Society angina classes III–IV were present in 46%. The majority of patients (64%) had single-vessel CAD while 34% had two-vessel and 2% had three-vessel involvement. The mean Logistic EuroSCORE I was 2.19 ± 1.53. Left internal mammary artery (LIMA) grafting was performed in 96% of cases. Additional conduits included left radial artery in 32% and saphenous vein in 8%, with T-grafts in 26% and sequential grafting in 4%. The average number of grafts per patient was 1.35 ± 0.53 (range 1–3). The procedure was performed off-pump in 96% of cases, with two patients (4%) requiring CPB support during conversion from mini-thoracotomy. The overall conversion rate to sternotomy was 16% (eight patients), predominantly due to difficult or injurious IMA harvest or anatomical limitations. The mean operative time was 197.8 ± 76.8 min and decreased significantly after the first 25 cases (220 min vs. 175 min). Atrial fibrillation occurred in 18%, pleural effusion in 28% (10% requiring drainage), and chest infection in 8%. Wound complications arose in 4%. There was no 30-day mortality. ICU stay averaged 2 ± 2.2 days (range 1–14), and total hospital stay was 5.7 ± 2.7 days where institutional coronary bypass stay is normally 7.9 +/− 7.0 days. Conclusion: These results demonstrate that MICS-OPCAB is a safe and feasible approach for selected patients requiring multivessel coronary artery bypass grafting. There are some technical challenges during the learning curve for which conversion to open surgery can confer good outcomes. Traversing the early learning curve can confer additional benefits to later patients.

## 1. Introduction

Coronary artery bypass grafting provides survival advantages over percutaneous intervention, as acknowledged by international guidelines [1,2]. Despite this, open cardiac surgery volumes have declined relative to interventional alternatives [3]. This trend, together with advances in surgical technology, has stimulated a shift toward less invasive approaches that aim to minimise operative trauma while maintaining the durability and completeness of revascularisation [3]. Minimally Invasive Direct Coronary Artery Bypass was the earliest iteration of this, allowing mammary harvest and single vessel coronary artery bypass grafting through a small mini-thoracotomy, the first description of which was for reoperative cases [4]. This allowed the left internal mammary to left anterior descending graft that was thought to be responsible for the primary survival advantage, but would lead to incomplete revascularisation in multivessel disease. Hybrid revascularisation, combining Minimally Invasive Direct Coronary Artery Bypass with percutaneous intervention (PCI) to the remaining territories has been posited. Immediate outcomes are favourable with a survival advantage to complete revascularisation over incomplete [5], but longer term (>2 y) freedom from reintervention compared to complete surgical revascularisation is not known [1]. A desire to see complete surgical revascularisation through minimally invasive methods was initially addressed with robotic approaches, but these require substantial capital investment and ongoing consumable and maintenance costs.

Minimally invasive off-pump coronary artery bypass grafting (MICS-OPCAB), typically performed via a left anterior mini-thoracotomy, has emerged as a cost-effective, safe, and effective alternative to conventional sternotomy, offering comparable graft patency and revascularisation outcomes while reducing surgical trauma and postoperative morbidity [1,5,6,7]. The procedure can be performed with predominantly conventional instruments and little additional equipment.

Contemporary evidence demonstrates that multivessel minimally invasive CABG via left anterior mini-thoracotomy achieves long-term graft durability equivalent to conventional sternotomy. In a large dual-centre series, five-year patency of the left internal mammary artery to the left anterior descending artery (LIMA–LAD) was comparable between MICS-OPCAB and standard CABG (92.1% vs. 93.4%, *p* = 0.38) [5]. Perioperative morbidity was also significantly reduced. In a propensity-matched study, MICS-OPCAB was associated with lower incidence of wound infections (0% vs. 4.0%, *p* = 0.03), decreased transfusion requirements (0.4 ± 1.2 vs. 1.8 ± 2.1 units, *p* = 0.003), and significantly shorter mechanical ventilation time (4.2 ± 3.1 vs. 12.5 ± 8.7 h, *p* < 0.001) compared with off-pump CABG via sternotomy [6,7].

We sought to examine our learning curve with this procedure and to identify the key challenges, pitfalls, and opportunities with introducing this technique to our centre.

## 2. Materials and Methods

### 2.1. Study Design and Setting

This retrospective single-operator study analysed 50 consecutive MICS-OPCAB procedures performed by one of the study authors (ADM) at Liverpool Heart & Chest Hospital NHS Foundation Trust. Cases were performed between January 2023 and June 2025, commencing with two proctored cases and transitioning to independent practice. The series represents the surgeon’s complete initial experience with the technique, from first adoption to procedural standardisation. The surgeon was an experienced off-pump coronary artery bypass grafting (OPCABG) surgeon prior to commencing MICS-OPCAB training. His OPCABG volume at the start of the programme was 945 OPCABG cases, with a conversion rate to on-pump of 1.1%. In order to familiarise himself with MICS-OPCAB techniques, he undertook four observerships at Milan, Dortmund, Dusseldorf, and Toronto, with the latter three including operating privileges allowing a more immersive educational experience.

### 2.2. Patient Selection

Patients were selected based on coronary anatomy suitability and those with target vessels without diffuse, intramyocardial, or heavily calcified coronary disease where the planned anastomotic site was perceived to be accessible through the utility incision (i.e., not too proximal). Additional selection criteria included body habitus (BMI < 30 initially) and excluding those with complex coronary anatomy (e.g., multiple sequential lesions in any target vessel), poor ventricular function, salvage and emergency procedures, and taking care with patients with pulmonary disease. Patients with borderline pulmonary function tests were trialled on single-lung ventilation prior to skin incision to ensure stability through the procedure though no patients needed to be converted to sternotomy for this reason.

### 2.3. Operative Technique

All procedures were performed using a standardised minimally invasive coronary artery bypass grafting (MICS-OPCAB) technique. The procedure is outlined in detail in the Supplement Materials. In brief, the procedure was performed with either double-lumen endotracheal intubation or a bronchial blocker for lung isolation, according to anaesthetist preference. A single mini-thoracotomy in the mid-clavicular line at the 5th intercostal space was used as a utility incision for mammary harvest under direct vision and all coronary grafting.

### 2.4. Outcome Measures

Outcome measures included complete revascularisation of all intended territories, in-hospital mortality, conversion to sternotomy, and perioperative morbidity. Length of hospital and ITU stay were also recorded.

### 2.5. Statistical Analysis

All analyses were performed using Microsoft Excel 365 (Microsoft Corporation, Redmond, WA, USA) supplemented with AI-guided commands to generate analytic formulas and Phantom scripting for graph generation. All outputs were manually verified.

Categorical variables are presented as frequencies (percentages), and continuous variables as mean ± Standard Deviation (SD) for normally distributed data, or median with interquartile range (IQR) for non-parametric data.

## 3. Results

### 3.1. Patient Demographics

Our study consisted of 50 patients with a mean age of 63.1 ± 8.7 years (range: 40–80), and a predominance of males (82%, *n* = 41). Patient characteristics at baseline are shown in Table 1. The average BMI was 27.8 ± 4.3 kg/m^2^ (range: 18.7–35.7), with 28% (*n* = 14) classified as normal weight, 40% (*n* = 20) overweight, and 32% (*n* = 16) obese; no patients were underweight. Hypertension was the most common comorbidity, present in 64% (*n* = 32), followed by diabetes mellitus in 26% (*n* = 13), and chronic kidney disease in 8% (*n* = 4). COPD was identified in 12% of patients (*n* = 6). A history of previous Myocardial Infarction was noted in 74% (*n* = 37), and 32% (*n* = 16) had undergone prior percutaneous coronary intervention (including one failed PCI). Regarding smoking history, 18% (*n* = 9) were active smokers and 30% (*n* = 15) were ex-smokers. According to the Canadian Cardiovascular Society (CCS) classification, 14% (*n* = 7) were in Class IV. Coronary angiography revealed single-vessel disease in 64% (*n* = 32), double-vessel disease in 34% (*n* = 17), and triple-vessel disease in 2% (*n* = 1). Preoperative risk assessment indicated a mean EuroSCORE of 2.19 ± 1.53 and a mean EuroSCORE II of 0.96 ± 0.36, reflecting a generally low- to moderate-risk population.

### 3.2. Operative Characteristics

Procedural success was assessed by complete revascularisation which was 100% with no in-hospital mortality. Quality measures for the approach were also recorded. A conversion to sternotomy occurred in 16% of cases (eight patients), largely driven by intraoperative factors such as complications in IMA harvesting (difficult or damaged IMA *n* = 4 (8%)), unfavourable anatomy (fused ribs *n* = 1 (2%)), or inadequate exposure of target vessels (*n* = 1 (2%). Additional conversions were necessary in response to intraoperative complications, including vessel injury or haemodynamic compromise *n* = 2 (4%), where continuing via the minimally invasive route was deemed unsuitable. Nonetheless, revascularisation was completed in all cases following conversion.

Secondary outcomes included re-explorations (4%), stroke (0%), wound infection (4%), and pneumonia (14%). Postoperative pleural effusions occurred in 28% of patients, with 10% requiring drainage and 18% successfully managed conservatively. The majority (72%) of patients did not develop any effusions. The median length of ICU stay was 1 day, (range 1–14, IQR 1–2), and the median hospital stay was 5 days (range 3–15, IQR 4–7). Average length of stay was 5.7 ± 2.7 days. Other complications recorded included episodes of postoperative atrial fibrillation, re-admissions, and mini-thoracotomy wound infections.

All procedures were performed through a mini-thoracotomy averaging 89 ± 12 mm. Off-pump surgery was the default strategy, achieved in 48 of 50 patients (96%); only two cases (4%) required unplanned cardiopulmonary bypass for haemodynamic instability. Single-vessel revascularisation predominated (CABG ×1: 32 cases, 64%) (Table 2), while 17 patients (34%) received two grafts and a single patient (2%) required three grafts. LIMA harvest was attempted in all patients and used in all but two patients where mid-vessel damage precluded its use. The only case in which the LIMA was not grafted to the LAD was where the true left anterior descending was recessive and small calibre and a parallel large diagonal was confluent, allowing superior run-off while still perfusing the anterior septum. The LIMA-LAD therefore was used in 96% of cases. Additional conduits included the left radial artery in 16 patients (32%)—frequently as a T-graft—and the saphenous vein in 4 patients (8%). In total, 86 distal anastomoses were fashioned (mean 1.35 per patient). Target-vessel distribution mirrored a focused, anterior strategy: the LAD was grafted in 98% of cases (96% LIMA and 2% radial), followed by diagonal/ramus branches (16%), obtuse marginal branches (12%), and the posterior descending artery (8%). Composite T-grafting was employed in 13 patients (26%) and sequential anastomoses in 2 (4%). Eight patients (16%) required conversion to sternotomy—most within the initial half of the series—and one patient who underwent a triple vessel MICS OPCAB (CABG ×3), anterior ECG, and TOE changes was noted immediately upon closure, before the patient left the operating theatre. On re-entry, a clot was found in the LIMA–LAD anastomosis, necessitating emergency sternotomy and conversion to cardiopulmonary bypass (CPB). All three grafts were revised as the extent of the LIMA thrombosis could not be guaranteed to exclude the composite. This approach contributed to the prolonged operative time of 487 min for this case. In a second patient, ST segment elevation was detected in the early postoperative period in the critical care unit (POCCU). Urgent angiography revealed a failed LIMA–LAD graft, and revascularisation was achieved via native LAD stenting. This was the only case in the series requiring postoperative percutaneous coronary intervention (PCI).

Mean ICU stay was 2.0 ± 2.2 days (range 1–14), despite a conversion rate typical of an early MICS experience. The patient who remained on critical care for 14 days was a conversion from MICS to open sternotomy for difficult access for IMA harvest. There were no in-hospital deaths.

### 3.3. Postoperative Outcomes

These are summarised in Table 3. Rhythm and respiratory complications were infrequent. New-onset atrial fibrillation (AF) occurred in 9 patients (18%), most AF episodes were transient; one patient required electrical cardioversion, and another was managed with oral anticoagulation (Apixaban). Seven patients (14%) developed a chest infection with all successfully treated with antibiotics. Pleural effusion occurred in 14 patients (28%); most (9/14) were managed conservatively, while 5 (12% of the entire cohort) required drainage. Eight patients (16%) were re-admitted within 30 days—5 for pleural effusion, one for elective cardioversion, one for pain evaluation with negative angiography, and one for combined effusion and mini-thoracotomy wound infection. No strokes, renal failures, or deep sternal wound infections were observed.

### 3.4. Operative Times and Learning Curve

**Figure 1:**
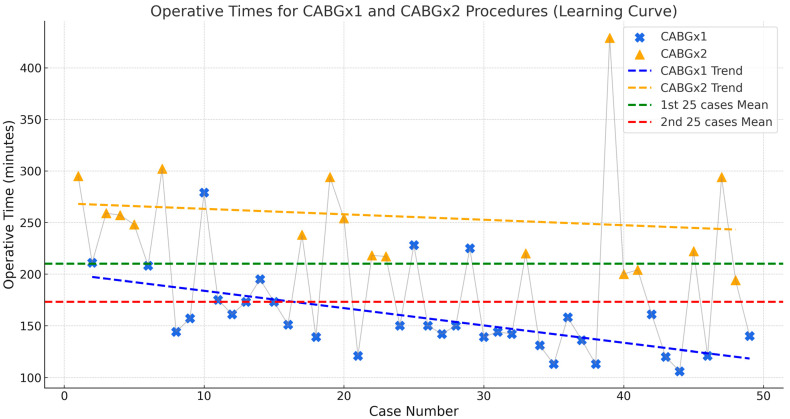
Learning curves for CABG ×1 and CABG ×2 procedures over the initial 50 MICS—OPCAB cases.

The mean operative time was 198 ± 77 min (range 106–487). Case-complexity strongly influenced duration (Figure 1).

- Single-graft cases: 158 ± 38 min (106–279);
- Double-graft cases: 256 ± 57 min (194–429).

When the series was split chronologically, mean operative time fell from 220 min in the first 25 cases to 175 min in the second 25, which was statistically significant at *p* = 0.037. This 20% reduction underscores a steep learning curve and rapid acclimatisation to the technique. (Figure 1).

- CABG ×1: Mean operative time: 158.0 ± 37.8 min (range: 106–279). A progressive decline in time is evident, reaching consistency after ~15 cases.
- CABG ×2: Mean operative time: 255.6 ± 55.3 min (range: 194–429). Despite greater complexity, operative duration decreased with experience.

Comparison of case halves:

- First 25 cases: Mean Op Time: 220.24 min. SD: 77.54 Range: 121–487 min;
- Second 25 cases: Mean Op Time: 175.28 min. SD: 70.50 Range: 106–429 min.

There is a clear reduction in operative time in the second half of cases (≈45 min improvement on average), suggesting a learning curve effect and increased proficiency with MICS-OPCAB as experience grew.

## 4. Discussion

This manuscript describes the initial stages of a single-centre multivessel minimally invasive coronary revascularisation programme. As far as we are aware there are no multi-centre or registry series in the literature. A recent systematic review has demonstrated that the procedure is concentrated at dedicated centres with no widespread adoption [8]. Whilst the cases have been chosen to try to maximise success, these data describe a process which has not been without difficulty. The senior author is an experienced OPCABG surgeon with extensive experience of multi- and total-arterial OPCABG surgery, and has been an OPCABG proctor for a decade. Despite this, the programme has been a challenge, but good results have been achieved.

An alternative for some of these patients could have been a robotically assisted MIDCAB procedure for which procedural times and costs are higher. Intensive care and hospital stay times compare favourably with sternotomy CABG cases in our institution with a reduced hospital stay compared to unselected CABG cases. Although we have not performed any formal health economic or cost-effectiveness calculations, it is very likely that this procedure provides superior value for money when in comparison with other techniques, even accounting for capital costs associated with the retractors and stabilising arms.

Institutional support is crucial in delivery of such a programme. Coronary artery bypass grafting is the most common cardiac surgery procedure performed in most European units. High volumes have conferred increasingly excellent results and the introduction of innovation is frequently fraught with uncertainty when the existing standard is so well performed. A strict process of governance and review is therefore required, with support to ensure that the challenges of the learning curve do not prematurely abort development. Patients must therefore be informed of the surgeon’s intention to undertake a novel procedure through a new access route that, while appealing, may be subject to higher risks. Our series demonstrates that those risks can be navigated safely.

This is a single-surgeon, single-institution experience, and so has all the limitations inherent with this type of experience, but it does indicate feasibility for such a programme. It is unlikely that this technique will be a solution for all patients requiring coronary artery intervention, but for those who are deemed to be appropriate, this method of revascularisation offers an attractive alternative to conventional CABG both on- and off-pump through a sternotomy.

Any single-individual venture has inherent risks that should that individual be unavailable, so a training programme for colleagues in our institution is planned, with two colleagues now learning these techniques. These colleagues are also experienced complex OPCABG surgeons, au fait with all of the requisite skills needed to allow proficiency with this procedure: total arterial revascularisation, composite grafting, and sequential anastomoses, which are mandatory for the approach utilised. It is envisaged that the learning curves of subsequent practitioners will be expedited by mutual support, shared learning, and in-parallel training to foster faster progress than if they were to train in series.

Prospective surgeons will need to be experienced coronary surgeons, and it is quite unlikely that a surgeon with little or no OPCABG experience would be successful with this procedure without an interim period of development in off-pump techniques. The limited access for grafting (even under favourable conditions) is very dissimilar to the wide access afforded by sternotomy and cardioplegic arrest.

## 5. Conclusions

This consecutive 50-patient experience demonstrates that a MICS-OPCAB programme can be established with low conversion to on-pump (4%), low revision and revascularisation rates (4%), and a rapid learning-curve-driven reduction in operative time. The data support expanding minimally invasive coronary surgery beyond single-vessel LAD grafting to selected multivessel cases while preserving excellent early outcomes.

## Figures and Tables

**Table 1:** Preoperative patient characteristics.

Preop Data (*n* = 50)	
Age, M ± SD & R	63.12 ± 8.7 (40–80)
Gender, Male *n* (%)	41 (82%)
BMI: M ± SD & R	27.8 ± 4.3|(18.7–25.7)
Underweight (<18.5): *n*	0
Normal (18.5–24.9): *n*	14
Overweight (25–29.9): *n*	20
Obese (≥30): *n*	16
Hypertension, *n* (%)	32 (64%)
Diabetes mellitus, *n* (%)	13 (26%)
Canadian Cardiovascular Society (CCS) grading	
Grade I	6 (12%)
Grade II	21 (42%)
Grade III	16 (32%)
Grade IV	7 (14%)
Smokers, *n* (%)	9 (18%), 15 ex-smokers (30%)
Chronic Kidney Disease, *n* (%)	4 (8%)
NSR (History), *n* (%)	48 (96%)
COPD, *n* (%)	6 (12%)
Thyroid disease	2 (4%)
Previous MI, *n* (%)	37 (74%)
Previous PCI, *n* (%)	16 (32%), including 1 failed PCI
CAD 1 Vessel, *n* (%)	32 (64%)
CAD 2 Vessel, *n* (%)	17 (34%)
CAD 3 Vessel, *n* (%)	1 (2%)
EuroScore, M ± SD	2.19 ± 1.53
EuroScore Median (IQR)	1.59 (1.16)
E2, M ± SD	0.96 ± 0.36
E2, Median (IQR)	0.88 (0.38)

M: mean; SD: standard deviation; R: Range, IQR: interquartile range, BMI: Body Mass Index, NSR: Normal Sinus Rhythm, MI: Myocardial Infarction, PCI: Percutaneous Coronary Intervention, CAD: Coronary Artery Disease.

**Table 2:** Operative details.

Operative Data	
Incision length, mm ± SD (mm)	89.2 ± 11.9 mm
Off-pump, *n* (%)	48 (96%)
Off-pump converted to On-pump, *n* (%)	2 (4%)
CABG ×1, *n* (% of total)	32 (64%)
CABG ×2, *n* (% of total)	17 (34%)
CABG ×3, *n* (% of total)	1 (2%)
Conduits	
LIMA, *n* (%)	48 (96%)
Left Radial Artery, *n* (%)	16 (32%)
SV, *n* (%)	4 (8%)
Grafts,	
Left Anterior Descending, *n* (%)	49 (98%)
Diagonal (D1, D2), *n* (%)	8 (16%)
Obtuse Marginal, *n* (%)	6 (12%)
Posterior Descending Artery, *n* (%)	4 (8%)
Ramus, *n* (%)	1 (2%)
Left Ventricular Branch, *n* (%)	1 (2%)
Grafts, M ± SD (R)	1.35 ± 0.53 (range 1–3)
T-Grafts, *n* (%)	18 (36%)
Sequential, *n* (%)	2 (4%)
Total anastomosis, *n*	86
Operation time, M ± SD (min) R	197.8 ± 76.8 min (106–487)
For single grafts M ± SD (min) R	158.0 ± 38.44 min (106–279)
For two grafts M ± SD (min) R	255.6 ± 57.0 min (194–429)
For three grafts (min)	487 min
Conversion to Sternotomy, *n* (%)	8 (16%)
Re-exploration, *n* (%)	1 (2%)
Graft revascularisation, *n* (%)	1 (2%)
Stent, *n* (%)	1 (2%)
ICU stay, M ± SD (Days)	2 ± 2.2 (range 1–14)
CKMB	
M ± SD & R	15.4 ± 27.9 (2–165) ng/mL
Median (IQR)	6 (4–11) ng/mL

mm: Millimetres, SD: standard deviation, *n*: number, R: range, CABG: coronary artery bypass grafts, CKMB: creatine kinase MB.

**Table 3:** Outcomes summary.

Postoperative Data	
Return to theatre, *n* (%)	2 (4%)
Post op AF (Any episode)	9 (18%)
Chest infections	7 (14%)
Stroke	0%
Pleural effusion, *n* (%)	14 (28%)
Managed conservatively, *n*	9
Drained, *n*	5
Mortality (intraop, 30-day)	0%
Hospital stay, M ± SD (Days)	5.7 ± 2.7 days (range 3–15)
Re-admissions *n* (%)	8 (16%)
For pleural effusion ± drain, *n*	5
AF (elective cardioversion), *n*	1
Pain work up (ruled out graft occlusion), *n*	1
Mini-thoracotomy wound infection, *n*	2

## Data Availability

The original contributions presented in this study are included in the article/Supplementary Materials. Further inquiries can be directed to the corresponding author(s).

## Supplementary Materials

The following supporting information can be downloaded at: https://www.mdpi.com/article/10.3390/jcdd12120456/s1, Figure S1: Thoracotomy exposure, pectoralis division using ThoraTrak™ retractor; Figure S2: Thoracotomy exposure, pectoralis division, and chest wall elevation using ThoraTrak™ retractor with ULTRAVISION™ CT Lift and hook; Figure S3: Blue arrow; Application of the Maquet Acrobat-i stabiliser with suction footplates either side of the LAD. Yellow arrow; Completed LIMA–LAD anastomosis under direct vision; Figure S4: Postoperative photograph demonstrating measurement of the thoracotomy scar. The intercostal drainage tube is seen positioned anteriorly on the chest. Reference [9] are cited in the Supplementary Materials.
