# Supplementary material for: Initial Outcomes from a Minimally Invasive Cardiac Surgery—Off-Pump Coronary Artery Bypass Grafting (MICS-OPCAB) Programme: A Case Series of the First 50 Patients Single-Centre Experience
Source: J Cardiovasc Dev Dis. 2025 Nov 25;12(12):456. doi: 10.3390/jcdd12120456 (PMC12734048; doi:10.3390/jcdd12120456)
Supplement: Supplementary file 1 [file jcdd-12-00456-s001.zip › jcdd-3934548 supplementary.pdf]

Supplement Materials

Surgical Access

Patients were placed in the supine position with a gel roll (Gel 'D' Pillow, Dublin: RYCOL Medical Ltd) cranio-caudally oriented under the left scapula to achieve modest thoracic elevation and expansion of the rib spaces on the left. Skin prep included to the left posterior axillary line and use of a fenestrated drape (Mölnlycke, Gothenburg, Germany) to include a window incorporating the midline in case of need for sternotomy and up to the mid-axillary line. The window on the drape was therefore typically placed obliquely across the patient.

A 7–10 cm left anterior minithoracotomy was performed in the 4th or 5th intercostal space, approximately 2–3 cm lateral to the sternal border (with approximately one third of the incision medial to the nipple) in males. In female patients, the incision was placed 5mm inferior to the infra-mammary crease (to avoid damaging breast tissue) in a similarly lateral position. Care was taken not to place the incision too close to the sternum, as this may risk inadvertent injury to the LIMA during intercostal entry.

The pectoralis major muscle was divided along the incision line to facilitate exposure. Chest wall retraction was achieved using the ThoraTrak™ retractor (Medtronic, Inc., Minneapolis, MN, USA (Figure S1)), in combination with the ULTRAVISION™ CT Lift IMA Exposure System and hook (TeDan Surgical Innovations, Inc., Texas, TX, USA). This setup provided stable rib elevation, allowing for excellent visualization of the internal mammary artery through a minimal-access approach. (Figure S2)

Conduit Harvesting

The left internal mammary artery (LIMA) was harvested as a pedicled graft under direct vision using extended-tip electrocautery and long Debakey forceps with standard

titanium clip applicators. If the applicator could not reach the apex for branch ligation, a long LigaClip™ multiple clip applicator (J&J MedTech, NJ, USA) was used. Heparin (300IU/kg) was administered prior to division of the LIMA aiming to have an ACT of >480s for the duration of grafting. The LIMA was clipped distally after checking flow and wrapped in papaverine-soaked gauze to prevent vasospasm.

If additional conduits were required:

· The radial artery (RA) (14% of cases) was harvested using an open approach (planned for future endoscopic harvest) after confirming adequate ulnar collateral flow with a modified Allen's test and Barbeau test with oximetry.

· A segment of the great saphenous vein (SVG) (8%) was harvested either endoscopically or via open technique.

· After retractor and stabilizer placement, the pericardium was opened longitudinally over the left ventricle, parallel to the delineation between the ventricles, which was typically evident following division and retraction laterally of the pericardial fatpad. This incision was then extended laterally from the inferior extent of the vertical incision into an 'L' shape, this being extended to the apex for multiple graft cases to allow access to the lateral and/or inferior surfaces. Pericardial stay sutures were placed on either side of the vertical pericardiotomy and both sides retracted laterally to enhance exposure of the target vessel(s) and minimize traction on surrounding structures.

Anaesthesia

Single-lung ventilation was facilitated using either a double-lumen endotracheal tube or a bronchial blocker depending upon anaesthetist preference. Bronchial blockers were typically preferred to obviate the need for tube exchange at the end of the case as we do

not routinely extubate on-table. Double-lumen tube was preferred to aid suction and lung deflation. Monitoring included arterial and central venous lines, transoesophageal echocardiography (TOE) for selected cases, and external defibrillator pads in the right axilla and posterior left chest.

Myocardial Stabilization & Protection

MICS off-pump beating heart CABG was the standard approach in our series.

The Octopus™ Nuvo tissue stabilizer (Medtronic, Inc., Minneapolis, MN, USA) or Maquet Acrobat i stabilizer (Getinge AB, Gothenburg, Sweden) was introduced typically through the thoracotomy to immobilize the target coronary segment. Depending on the access with the device through the incision, an Iron Assistant TM (Geister, GmbH, Germany) was placed through a medial port incision that traversed the same intercostal space as the mini-thoracotomy (to reduce the number of neurovascular bundles involved). Suction footplates were applied to stabilize the LAD. Intracoronary shunting was used routinely to maintain distal perfusion. (Figure S3)

Access to the lateral and inferior walls was achieved by using a Starfish positioner (Medtronic, Inc., Minneapolis, MN, USA). This was detached from its armature, a robust suture tied to its base and the device then applied to the heart to allow retraction to expose whichever artery was the grafting target. Usually, the Starfish was placed on the oblique margin for lateral grafting and the inferior aspect of the apex for inferior grafting.

Anastomosis

The LIMA–LAD anastomosis was completed under direct vision using continuous 7-0 polypropylene suture. (Figure S3) The surgeon’s technique for LIMA-LAD was identical to his open technique aside from performing the anastomosis in an anti-clockwise fashion rather than his usual clockwise, as this was easier due to access limitations. To perform

this, the LIMA was rotated 180 degrees so that the lumen was uppermost. A 7/0 prolene suture was placed from within the LIMA heel on the caudal side to outside and the suture end secured with a light bulldog clip. The other end of the suture was then passed from inside the LAD heel on the lateral side, before continuing the suture anticlockwise. After three heel sutures, the LIMA was parachuted onto the anastamosis and the suturing completed. The surgeon stood on the patient's left for this anastomosis. For lateral grafting, the surgeon typically stood on the patient's right, the anastomosis being constructed identically to his open technique (starting at the medial aspect of the heel and passing clockwise). For inferior grafts, the surgeon stood on either side of the patient (depending upon access) with the anastomotic technique being identical to his open technique here also (starting at the right side of the heel and moving clockwise to the toe, before changing suture arm and coming back up to the toe from the heel).

The majority of patients (96%) underwent successful revascularization without the need for cardiopulmonary bypass (CPB). Only 2 out of 50 patients (4%) required conversion to CPB. In both cases, CPB was instituted emergently following CPR due to intraoperative complications: one patient developed VFib while awaiting a suitable intracoronary shunt, and another had an acute intraoperative graft thrombosis necessitating revascularization under bypass. Both patients survived. These instances highlight the feasibility of MICS-OPCAB approach, with CPB reserved for bailout scenarios.

Extrapleural analgesia was administered intraoperatively prior to chest closure, with the chest still open. A catheter-based left-sided extrapleural block of 0.25% levobupivicaine (maximum dose 0.8ml/kg) was placed under direct vision. This was placed in the extra-

pleural space as posteriorally as possible in the same rib space as the incision Subsequently, an infusion of 0.1-0.2 ml/kg/hr of 0.125% levobupivicaine was administered for a period of 2-3 days depending upon the patient. This approach provided targeted analgesia, facilitated early extubation, and reduced postoperative opioid requirements. [9].

The thoracotomy was closed by approximating the rib space with 5-metric Ethibond pericostal sutures.. The chest wall musculature was reapproximated in layers in a standard continuous fashion. A single chest drain was routinely inserted medial to the incision but in the same dermatome and passing through the same intercostal space. (Figure S4)

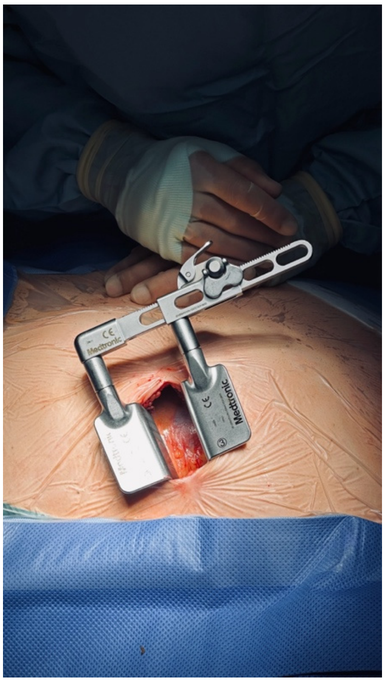

Figure S1 Thoracotomy exposure, pectoralis division using ThoraTrak™ retractor.

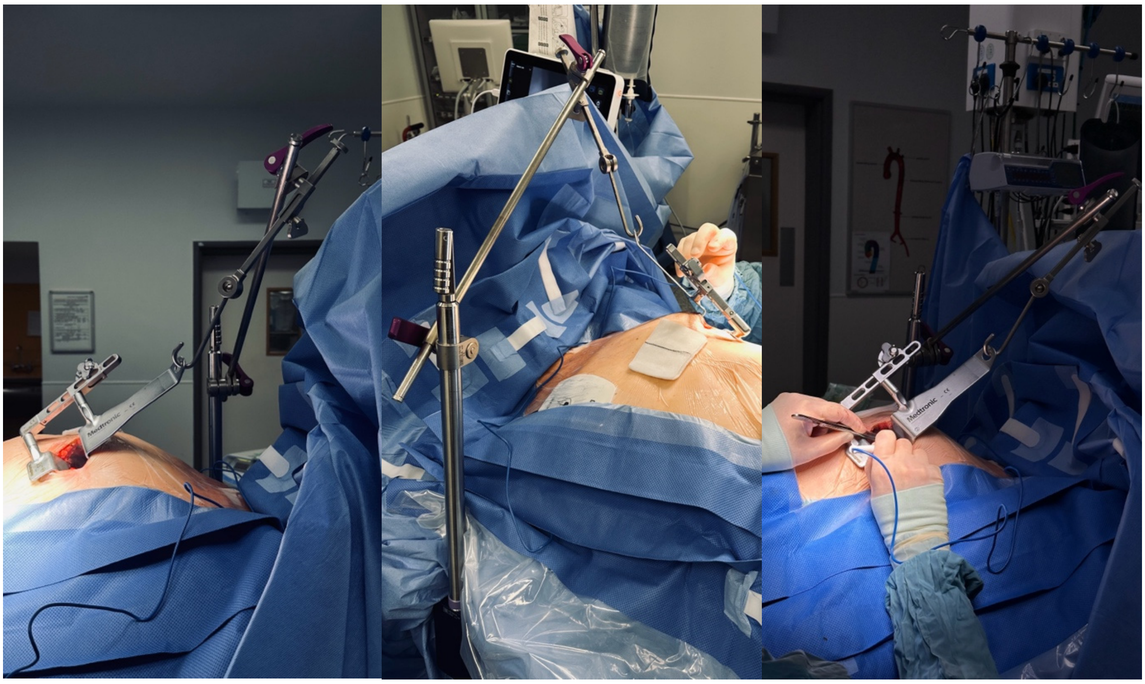

Figure S2: Thoracotomy exposure, pectoralis division, and chest wall elevation using ThoraTrak™ retractor with ULTRAVISION™ CT Lift and hook.

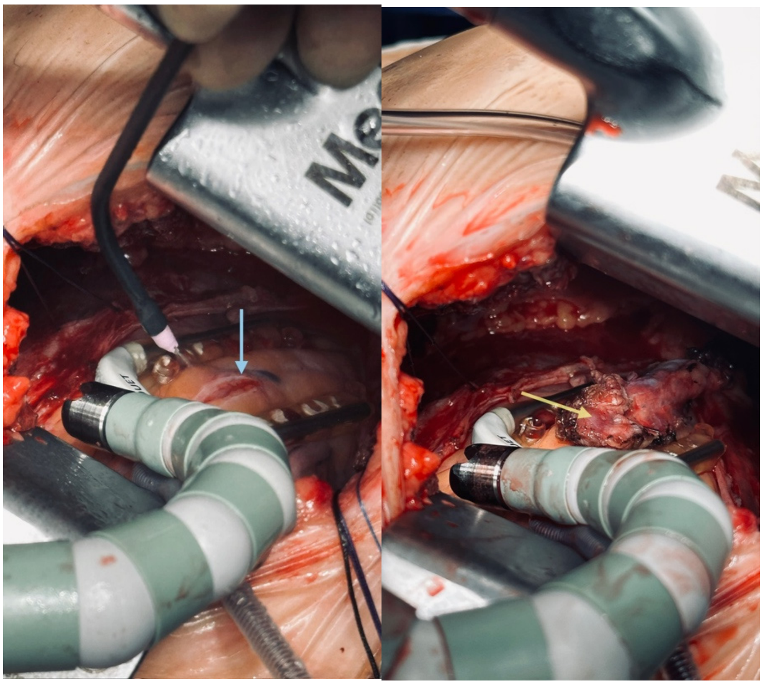

Figure S3:
Blue arrow; Application of the Maquet Acrobat-i stabilizer with suction footplates either side of the LAD.
Yellow arrow; Completed LIMA–LAD anastomosis under direct vision.

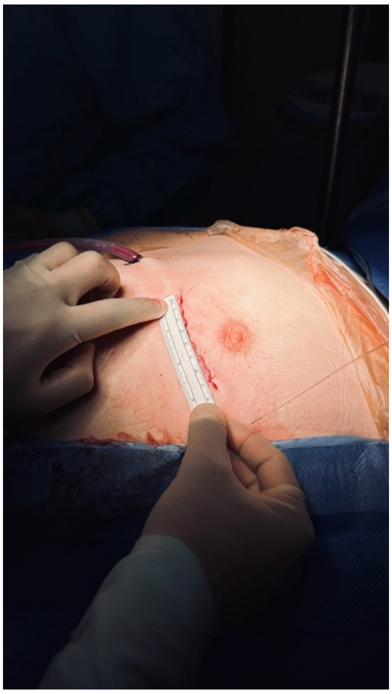

Figure S4:

Postoperative photograph demonstrating measurement of the thoracotomy scar. The intercostal drainage tube is seen positioned anteriorly on the chest.
